# P-1708. Identification and Analysis of Asymptomatic Pathogen Carriage in Stool Donors for Manufacturing of the Microbiome Therapeutic, Fecal Microbiota Spores, live-brpk

**DOI:** 10.1093/ofid/ofaf695.1880

**Published:** 2026-01-11

**Authors:** Sarah Tomkovich, Allison Nelson, Ketankumar Patel, Nicole Delehanty, Trudi Delk, Maximillian von Eynatten, Jason Goldsmith

**Affiliations:** Nestlé Health Science, Bridgewater, New Jersey; Nestlé Health Science, Bridgewater, New Jersey; Nestlé Health Science, Bridgewater, New Jersey; Nestlé Health Science, Bridgewater, New Jersey; Nestlé Health Science, Bridgewater, New Jersey; Nestlé Health Science, Bridgewater, New Jersey; Nestlé Health Science, Bridgewater, New Jersey

## Abstract

**Background:**

Fecal microbiota spores, live-brpk (VOWST®; formerly SER-109, hereafter VOS), is an oral microbiome therapeutic used to prevent recurrent Clostridioides difficile infection (rCDI) in adults post-antibiotic treatment. Derived from donor stool, donor recruitment, screening, and retention are crucial to manufacturing and safety. Multiple donations from a single donor make-up a drug lot, enabling monitoring of asymptomatic pathogen carriage rates in healthy donors.

**Figure 1.**
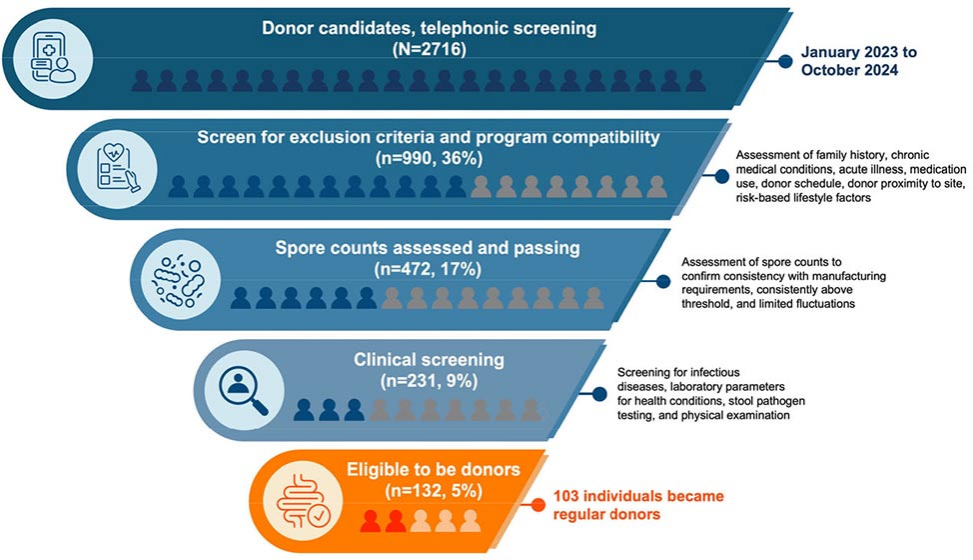
Multistep donor candidate screening for manufacturing of VOS. VOS, fecal microbiota spores, live-brpk (formerly SER-109).

**Table 1.**
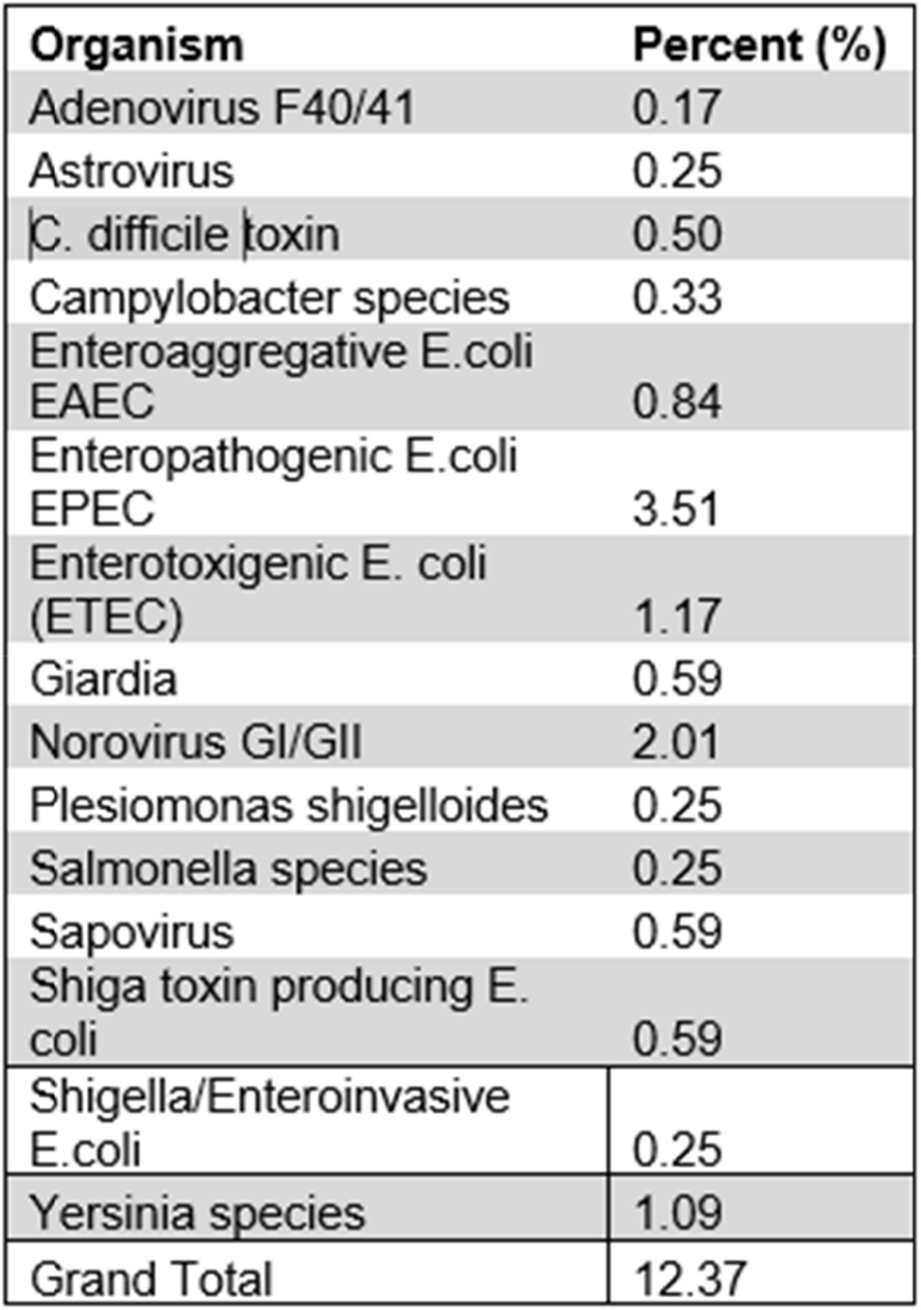
Gastrointestinal Panel Pathogen Positivity, Sept23-Oct24

**Methods:**

Donor candidates are initially screened by phone for the VOS program. Those who pass then undergo in-person clinical screening. Candidates who do not meet the requirements are deferred temporarily or permanently from the program due to reasons like infectious diseases, chronic conditions, risky travel, or microbiome-impacting medication. Analysis included donor demographics, visits, deferral reasons, clinical criteria, and stool spore counts from January 2023 to October 2024. Infectious disease screening results from September 2023 to October 2024 were used to identify asymptomatic carriage rates.

**Results:**

A total of 2716 donor candidates were assessed for program compatibility via telephonic screening (Figure 1); however, through subsequent attrition only 103 individuals became regular donors. During the subperiod of Sept ‘23-Oct ’24, 1196 GI pathogen panels were performed, with a total positively of 12.4% (Table 1). Only 8 tests were performed in donors with symptoms of diarrhea, and only 1 of those specimens was positive GI pathogens. Thus, there appears to be a ∼12% asymptomatic carriage rate across the various organisms, the most common being Enteropathogenic E. coli (EPEC, 3.51%) Norovirus GI/GII (2.01%, with reports of high false positives by the vendor), and Yersinia species (1.09%). Only 16 of 1659 COVID samples tested positive (0.96%) during this time.

**Conclusion:**

The robust clinical screening procedures provide an opportunity to appreciate the asymptomatic carriage rate of various pathogens in the healthy donor population. These data suggest a higher carriage rate than previously described for many organisms and demonstrates the importance of periodic, robust screening and manufacturing controls for donor-derived microbiome therapeutics.

**Disclosures:**

Sarah Tomkovich, PhD, Nestlé Health Science: Employee and May own stock Allison Nelson, n/a, Nestlé Health Science: Employee and May own stock Ketankumar Patel, MD, MBA, Nestlé Health Science: Employee and May own stock Nicole Delehanty, DNP, Nestlé Health Science: Employee and May own stock Trudi Delk, PharmD, Nestlé Health Science: Employee and May own stock Maximillian von Eynatten, MD, Nestlé Health Science: Employee and May own stock Jason Goldsmith, MD, PhD, Nestlé Health Science: Employee and May own stock

